# Triglyceride/low-density-lipoprotein cholesterol ratio is the most valuable predictor for increased small, dense LDL in type 2 diabetes patients

**DOI:** 10.1186/s12944-021-01612-8

**Published:** 2022-01-07

**Authors:** Gen Ouchi, Ichiro Komiya, Shinichiro Taira, Tamio Wakugami, Yusuke Ohya

**Affiliations:** 1 Department of Emergency and Critical Care Medicine, University of the Ryukyus Hospital, 207 Uehara, Nishihara, Okinawa 903-0215 Japan; 2 Department of Internal Medicine, Okinawa Medical Hospital, 2310 Tsuhako-Nishihara, Sashiki, Nanjo, Okinawa 901-1414 Japan; 3 Department of Diabetes and Endocrinology, Medical Plaza Daido Central, 123 Daido, Naha, Okinawa 902-0066 Japan; 4 Department of Internal Medicine, Okinawa Rehabilitation Center Hospital, 2-15-1 Hiyane, Awase, Okinawa, Okinawa 904-2173 Japan; 5 Department of Cardiology, Neurology and Nephrology, University of the Ryukyus Hospital, 207 Uehara, Nishihara, Okinawa 903-0215 Japan

**Keywords:** TG/LDL-C ratio, Non–HDL-C, Small, Dense LDL, Triglycerides, Type 2 diabetes mellitus

## Abstract

**Background:**

Small, dense low-density lipoprotein (sd-LDL) increases in type 2 diabetes patients and causes arteriosclerosis. Non–high-density-lipoprotein cholesterol (non–HDL-C) is thought to be useful for predicting arteriosclerosis and sd-LDL elevation; however, there are no data about whether the triglyceride /low-density-lipoprotein cholesterol (TG/LDL-C) ratio is a valuable predictor for sd-LDL.

**Methods:**

A total of 110 type 2 diabetes patients with hypertriglyceridemia were analyzed. No patients were treated with fibrates, but 47 patients were treated with statins. LDL-C was measured by the direct method. LDL-migration index (LDL-MI) using electrophoresis (polyacrylamide gel, PAG) was calculated, and a value ≥0.400 was determined to indicate an increase in sd-LDL. Simple regression analyses were carried out between LDL-MI and lipid markers. Receiver operating characteristic curves of lipid markers for predicting high LDL-MI were applied to determine the area under the curve (AUC), sensitivity, specificity, and cut-off point.

**Results:**

LDL-MI correlated negatively with LDL-C (*P* = 0.0027) and PAG LDL fraction (*P* < 0.0001) and correlated positively with TGs, non–HDL-C, TG/LDL-C ratio, TG/HDL-C ratio, and non–HDL-C/HDL-C ratio among all study patients. Similar results were obtained for patients analyzed according to statin treatment. The AUCs (95% confidence interval) were 0.945 (0.884-1.000) for TG/LDL-C ratio and 0.614 (0.463-0.765) for non–HDL-C in patients without statins (*P* = 0.0002). The AUCs were 0.697 (0.507-0.887) for TG/LDL-C and 0.682 (0.500-0.863) for non–HDL-C in patients treated with statins. The optimal cut-off point for TG/LDL-C ratio for increased LDL-MI was 1.1 (molar ratio) regardless of statin treatment. The sensitivity and specificity of the TG/LDL-C ratio (90.0 and 93.9%, respectively) were higher than those of non–HDL-C (56.7 and 78.8%, respectively) in patients without statins.

**Conclusions:**

The TG/LDL-C ratio is a reliable surrogate lipid marker of sd-LDL and superior to non–HDL-C in type 2 diabetes patients not treated with statins.

## Introduction

The risk of cardiovascular disease (CVD) is reportedly associated with an increase in small, dense low-density lipoprotein (sd-LDL) levels rather than large, buoyant LDL [1]. Sd-LDL reportedly exhibits several potentially atherogenic properties, such as reduced receptor-mediated clearance, prolonged retention in circulation, greater arterial wall retention, and increased oxidation [2]. Japanese elderly men with ischemic heart disease and high sd-LDL were shown to have increased risk of CVD events over the next 5 years [3]. Higher sd-LDL occurs when both non–high-density-lipoprotein cholesterol (non–HDL-C) and triglycerides (TGs) are high [4]. Non–HDL-C, having higher atherosclerosis-inducing properties than LDL-cholesterol (LDL-C), is increased in hypertriglyceridemia [5] and associated with increased sd-LDL [6]. According to current guidelines for arteriosclerosis, non–HDL-C should be evaluated instead of LDL-C in cases such as severe hypertriglyceridemia [7]. When the non–HDL-C level is high, cholesterol-lowering therapy is prioritized, as in LDL cholestrolemia [7, 8]. When the TG level is ≥4.5 mmol/L, Friedewald’s formula cannot accurately evaluate LDL-C, so non–HDL-C is used as a marker instead of LDL-C.

A recent study evaluating the effect of pemafibrate on hypertriglyceridemia in type 2 diabetes reported that baseline LDL-C (measured via the direct method) and LDL fraction (polyacrylamide gel [PAG] electrophoresis) decreased with increasing baseline sd-LDL [9]. Pemafibrate, a selective peroxisome proliferator-activated receptor alpha modulator, allows control of serum TG levels and sd-LDL, which were previously inadequate with conventional treatment [10, 11]. In more than half of type 2 diabetes patients with hypertriglyceridemia, baseline LDL-C was relatively lower than after pemafibrate administration [9]. Higher baseline TG level also appears to be involved in the baseline sd-LDL increase and post-dose LDL-C increase. In untreated type 2 diabetes patients with hypertriglyceridemia, large, buoyant LDL was reduced, suggesting that it accounts for low serum LDL-C measurements.

The change in non–HDL-C may not accurately reflect the change in LDL composition; that is, if the increase in TGs and decrease in LDL (mainly large, buoyant LDL) occur synchronously, non–HDL-C does not change markedly. This was shown in a previous study examining the effect of pemafibrate [9]. Diabetes patients with high TG but normal or low LDL-C may have higher sd-LDL. It was hypothesized that the TG/LDL-C ratio would be useful for predicting higher sd-LDL or apolipoprotein-B (apoB)-containing lipoproteins as an alternative to non–HDL-C. The present study clarified whether the TG/LDL-C ratio is more valuable than non–HDL-C or other lipid markers in predicting sd-LDL level in type 2 diabetes patients treated with or without statins.

## Materials and methods

### Patients and study procedures

**Fig. 1:**
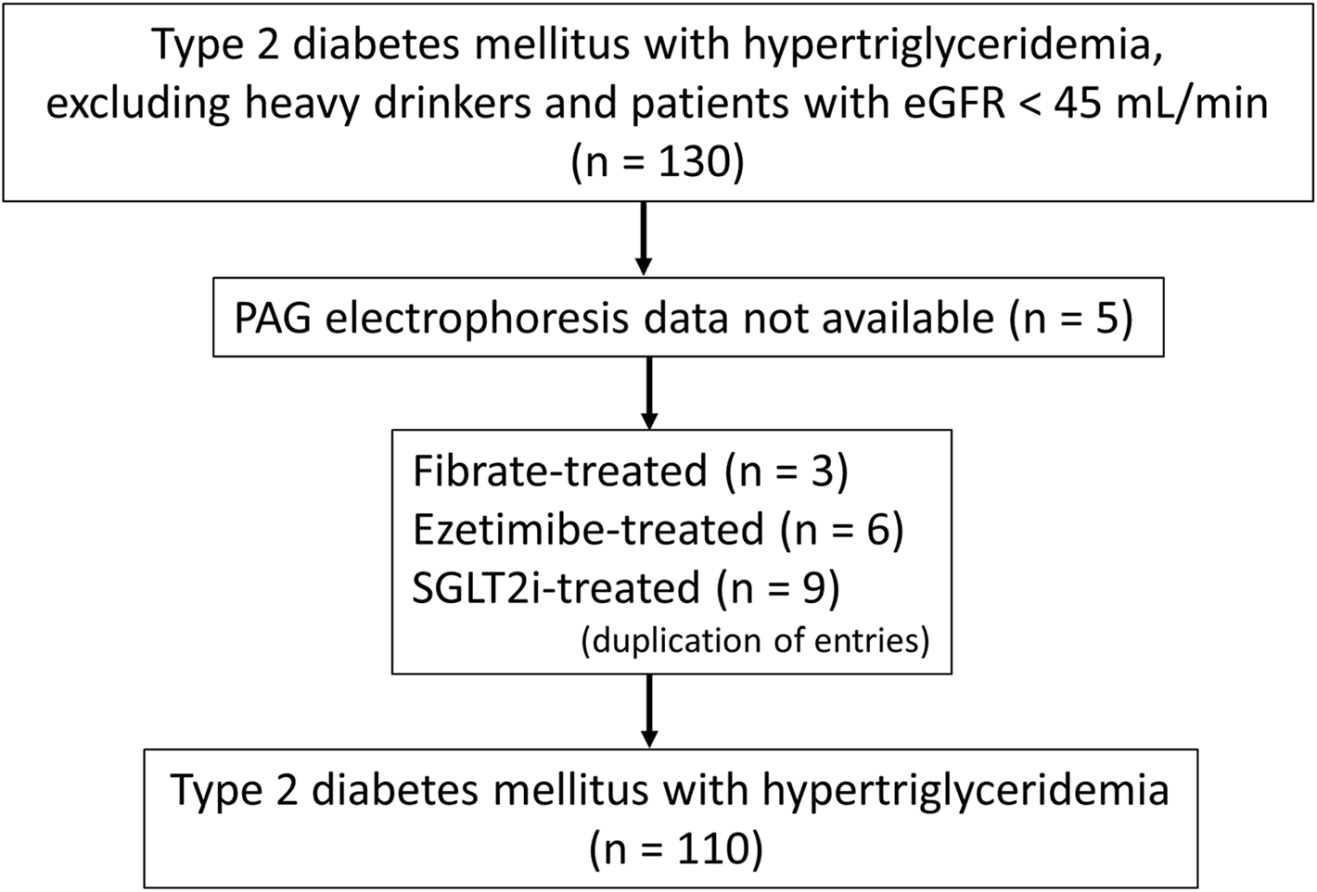
Case-finding protocol. eGFR, estimated glomerular filtration rate; PAG, polyacrylamide gel; SGLT2i, sodium-glucose cotransporter 2 inhibitors

Subjects in this retrospective study were outpatients with type 2 diabetes visiting the Medical Plaza Daido Central. A total of 130 patients with type 2 diabetes with hypertriglyceridemia were enrolled in this study after excluding heavy drinkers and patients with an estimated glomerular filtration rate (eGFR) < 45 mL/min. Five patients were excluded because PAG electrophoresis data were not available. Fifteen patients treated with conventional fibrates, ezetimibe, or sodium-glucose cotransporter 2 inhibitors were also excluded. The final study group consisted of 110 patients, including 47 patients treated with statins and 63 not treated with statins (Fig. 1). Because TG levels tend to fluctuate under the influence of diet [12], it was confirmed that fasting TGs were ≥ 1.7 mmol/L (150 mg/dL) by repeated measurements in the 110 diabetes patients enrolled in the study. The average of 2 or 3 measurements was used as the baseline value. Blood samples were collected after 9-12 h of fasting. All 110 patients are still being followed, and more than half of them have been treated with fibrates. In this study, all baseline data were analyzed prior to intervention with fibrates.

Lipoprotein electrophoresis (PAG) was conducted in all patients. PAG electrophoresis revealed 4 lipoprotein fractions (HDL, LDL, midband, and very-low-density lipoprotein [VLDL]). The LDL-migration index (LDL-MI) was calculated from the pattern of PAG electrophoresis according to a previous report [13, 14]; that is, the PAG electrophoretic distance between the LDL and VLDL fractions was divided by that between the HDL and VLDL fractions. When this value was ≥0.400, it was determined to indicate an increase in sd-LDL [13, 14]. LDL-C was measured via the direct homogenized method [15, 16]. If the TG level was 11.3 mmol/L (1,000 mg/dL) or higher, the LDL-C level measured by the direct method would be unreliable, but there were no such subjects.

### Statistical analysis

Data are shown as the mean ± SD (normal distribution), median (interquartile range [IQR]) (nonparametric distribution), or percentage. Results of TG and TG-related variable analyses are shown as the median (25-75% quartiles, IQR) due to non-parametric distribution. Comparisons between groups were made using *t*-test, Mann-Whitney *U* test, or the *χ*^*2*^ test. A simple regression analysis using the least squares method was applied for continuous variables (TGs, LDL-C, non − HDL-C, TG/LDL-C ratio, TG/HDL-C ratio, LDL/HDL-C ratio, non–HDL-C/HDL-C ratio, LDL, midband, and VLDL fractions in PAG) as explanatory variables and with LDL-MI as a response variable to determine the regression coefficient (*r*), standard error (SE), 95% confidence interval (CI), and standardized *R*^*2*^ values. LDL-MI ≥ 0.400 was predictive of higher sd-LDL level. A receiver operating characteristic (ROC) curve was generated to evaluate the discriminatory ability of the variables for higher LDL-MI, and the area under the curve (AUC) with its 95% CI was calculated. To determine the optimal cut-off points, sensitivity, and specificity of LDL-C, TGs, non–HDL-C, LDL and midband fractions in PAG, TG/LDL-C ratio, TG/HDL-C ratio, LDL-C/HDL-C ratio, and non–HDL-C/HDL-C ratio, the square root of ([1 − sensitivity]^2^ + [1 − specificity]^2^) was calculated, which represented the point on the ROC curve with the shortest distance from the upper left corner.

JMP for Windows software, version 12 (SAS Institute Japan; Tokyo, Japan), was used for statistical analyses. ROC analyses were performed using EZR (Saitama Medical Center, Jichi Medical University, Saitama, Japan), a graphical user interface for R (The R Foundation for Statistical Computing, Vienna, Austria) [17]. *P* values of < 0.05 were considered statistically significant.

## Results

### Comparison of clinical parameters in type 2 diabetes patients with hypertriglyceridemia

The clinical parameters of 110 type 2 diabetes patients are shown in Table 1. The proportion of males was high. Mean body mass index was 26.7 (SD 3.9) kg/m^2^. The average glycated hemoglobin was 7.3 (1.4)%, and the average eGFR was 72.4 (18.8) mL/min/1.73 m^2^. Hypertension and CVD/stroke complications were reported for 67.3 and 19.1% of patients, respectively. Comparing patients with and without statin treatment, the mean age was higher in patients treated with statins (66.6 [9.8] years) than in those not treated with statins (59.4 [13.6] years) (*P* = 0.0027, *t*-test), and the average eGFR was lower in patients treated with statins (66.6 [13.4] mL/min/1.73 m^2^) than in those not treated with statins (76.7 [21.1] mL/min/1.73 m^2^) (*P* = 0.0051). However, there were no significant differences in other clinical parameters between the two groups. With regard to lipid markers, patients treated with statins were characterized by lower TGs, higher HDL-C, and lower non–HDL-C levels compared with patients not treated with statins. Patients treated with statins were characterized by lower TG/HDL-C and non–HDL-C/HDL-C ratios compared with patients not treated with statins (Table 1).

**Table 1:** Characteristics of type 2 diabetes patients with hypertriglyceridemia

Variables	Total	With statins	Without statins	P*
Number (%)	110	47	63	
Age, mean ± SD, years	62.5 ± 12.6	66.6 ± 9.8	59.4 ± 13.6	0.0027
Men: Women	70: 40	26: 21	44: 19	0.1173
BMI, mean ± SD, kg/m^2^	26.7 ± 3.9	26.8 ± 4.3	26.6 ± 3.6	0.7968
HbA1c, mean ± SD, %	7.3 ± 1.4	7.4 ± 1.4	7.2 ± 1.5	0.6683
eGFR, mean ± SD, mL /min/1.73 m^2^	72.4 ± 18.8	66.6 ± 13.4	76.7 ± 21.1	0.0051
Complications				
Hypertension, no. (%)	74 (67.3)	34 (72.3)	40 (63.5)	0.3279
CVD/stroke, no. (%)	21 (19.1)	9 (19.2)	12 (19.1)	0.9893
Treatment for diabetes				
OHA, no. (%)	60 (54.5)	30 (63.8)	30 (47.6)	0.2835
Insulin + OHA, no. (%)	16 (14.5)	7 (15.0)	9 (14.3)	
Insulin, no (%)	4 (3.6)	1 (2.1)	3 (4.8)	
TG, median (IQR), mmol/L	3.1 (2.4-4.2)	2.9 (2.2-3.5)	3.3 (2.5-5.3)	0.0167
LDL-C, mean ± SD, mmol/L	2.9 ± 0.9	2.9 ± 0.7	2.9 ± 1.0	0.9221
HDL-C, mean ± SD, mmol/L	1.2 ± 0.3	1.3 ± 0.3	1.2 ± 0.3	0.0150
Non − HDL-C, mean ± SD, mmol/L	4.6 ± 1.0	4.3 ± 0.8	4.8 ± 1.1	0.0122
TG/LDL-C ratio, median (IQR)	1.0 (0.8-1.7)	0.9 (0.7-1.3)	1.1 (0.8-2.2)	0.0803
TG/HDL-C ratio, median (IQR)	2.5 (1.8-3.0)	2.3 (1.5-3.1)	2.7 (1.9-5.4)	0.0169
LDL-C/HDL-C ratio, mean ± SD	2.5 ± 0.9	2.3 ± 0.8	2.6 ± 0.9	0.1050
Non − HDL-C/HDL-C ratio, mean ± SD	4.1 ± 1.8	3.5 ± 1.3	4.5 ± 2.0	0.0039
Lipoprotein fraction (PAG electrophoresis)				
HDL, mean ± SD, %	20.7 ± 5.6	21.4 ± 4.5	20.2 ± 6.4	0.2620
LDL, mean ± SD, %	39.7 ± 12.7	41.7 ± 10.0	38.2 ± 14.3	0.1544
Midband, mean ± SD, %	17.6 ± 8.5	17.7 ± 7.8	17.5 ± 9.0	0.9419
VLDL, mean ± SD, %	20.1 ± 8.4	19.1 ± 6.5	24.1 ± 9.1	0.0023
LDL-MI, median (IQR)	0.393 (0.353-0.420)	0.381 (0.350-0.404)	0.398 (0.355-0.426)	0.0781

*P, t-test or Mann-Whitney U test between patients with and without statins

*BMI* body mass index, *HbA1c* glycated hemoglobin, *eGFR* estimated glomerular filtration rate, *CVD* cardiovascular diseases, *OHA* oral hypoglycemic agents, *TG* triglycerides, *LDL-C* low-density lipoprotein cholesterol, *Non − HDL-C* non − high-density lipoprotein cholesterol, *HDL-C* high-density lipoprotein cholesterol, *LDL-MI* LDL migration index, *PAG* polyacrylamide gel.

Table 1 also shows the results of lipoprotein fraction analyses and LDL-MI in PAG electrophoresis. PAG electrophoresis revealed 4 lipoprotein fractions (HDL, LDL, midband, and VLDL), but there were cases in which the midband fraction was not present. The VLDL fraction was lower in patients treated with statins than in those not treated with statins (19.3 [SD 6.5]% vs. 24.1 [9.1]%, *P* = 0.0023). There was no statistically significant difference in LDL fraction between patients treated with statins and those not treated with statins (41.7 [10.0]% vs. 38.3 [14.3]%, *P* = 0.1544). The LDL-MI in patients treated with statins was 0.381 (IQR 0.350-0.404), which was lower than that in patients not treated with statins (0.498 [0.355-0.426]), but the difference was not statistically significant (*P* = 0.0781).

### Simple regression analyses between lipid markers and LDL-MI

In simple regression correlation analyses of the 110 patients, LDL-MI levels correlated with 10 lipid markers, except LDL-C/HDL-C ratio (Table 2). An inverse correlation was observed between LDL-MI and LDL fraction (PAG) (*R*^2^ = 0.2396, *P* < 0.0001, least squares) and LDL-MI and LDL-C (*R*^2^ = 0.0804, *P* = 0.0027). Positive correlations were observed between LDL-MI and the other 7 lipid markers. When patients were analyzed based on statin treatment status, an inverse correlation was observed between LDL-MI and LDL fraction in patients treated with statins (*R*^2^ = 0.1351, *P* = 0.0113) and in those not treated with statins (*R*^2^ = 0.2668, *P* < 0.0001). Results similar to those obtained for all study patients were observed when examining the relationship between the other 9 lipid markers and LDL-MI according to statin treatment status.

**Table 2:** Simple regression analysis between LDL-MI and lipid markers in 110 type 2 diabetes patients with hypertriglyceridemia

Variables	r	SE	95% CI		P*	Adjusted R^2^
			Lower	Upper		
**Total**						
TG, mmol/L	0.0188	0.0021	0.0147	0.0229	< 0.0001	0.4429
LDL-C, mmol/L	− 0.0183	0.0060	−0.0301	−0.0065	0.0027	0.0804
Non − HDL-C, mmol/L	0.0207	0.0050	0.0108	0.0307	< 0.0001	0.1359
TG/LDL-C ratio	0.0222	0.0030	0.0161	0.0282	< 0.0001	0.3299
TG/HDL-C ratio	0.0109	0.0015	0.0080	0.0139	< 0.0001	0.3390
LDL-C/HDL-C ratio	0.0019	0.0063	−0.0105	0.0144	0.7572	0.0009
Non − HDL-C/HDL-C ratio	0.0150	0.0027	0.0097	0.0203	< 0.0001	0.2252
LDL (PAG), %	−0.0021	0.0004	−0.0029	−0.0014	< 0.0001	0.2396
Midband (PAG), %	0.0031	0.0006	0.0020	0.0042	< 0.0001	0.2308
VLDL (PAG), %	0.0024	0.0006	0.0012	0.0036	0.0001	0.1309
**With statins**						
TG, mmol/L	0.0156	0.0033	0.0089	0.0222	< 0.0001	0.3314
LDL-C, mmol/L	−0.0077	0.0094	−0.0266	0.0113	0.4203	0.0149
Non − HDL-C, mmol/L	0.0227	0.0072	0.0083	0.0372	0.0027	0.1826
TG/LDL-C ratio	0.0167	0.0045	0.0077	0.0258	0.0006	0.2349
TG/HDL-C ratio	0.0113	0.0027	0.0059	0.0167	0.0001	0.2825
LDL-C/HDL-C ratio	0.0049	0.0085	−0.0122	0.0220	0.5687	0.0073
Non − HDL-C/HDL-C ratio	0.0152	0.0045	0.0061	0.0243	0.0015	0.2018
LDL (PAG), %	−0.0016	0.0006	−0.0028	−0.0004	0.0110	0.1351
Midband (PAG), %	0.0033	0.0007	0.0020	0.0046	< 0.0001	0.3588
VLDL (PAG), %	0.0007	0.0010	−0.0013	0.0026	0.4999	0.0102
**Without statins**						
TG, mmol/L	0.0199	0.0028	0.0145	0.0254	< 0.0001	0.4520
LDL-C, mmol/L	−0.0222	0.0076	−0.0374	−0.0070	0.0050	0.1222
Non − HDL-C, mmol/L	0.0181	0.0071	0.0038	0.0323	0.0139	0.0953
TG/LDL-C ratio	0.0240	0.0042	0.0157	0.0323	< 0.0001	0.3542
TG/HDL-C ratio	0.0106	0.0019	0.0068	0.0145	< 0.0001	0.3328
LDL-C/HDL-C ratio	0.0022	0.0088	−0.0198	0.0153	0.7994	0.0011
Non − HDL-C/HDL-C ratio	0.0143	0.0036	0.0071	0.0216	0.0002	0.2033
LDL (PAG), %	−0.0022	0.0005	−0.0032	−0.0013	< 0.0001	0.2668
Midband (PAG), %	0.0031	0.0008	0.0015	0.0047	0.0003	0.1975
VLDL (PAG), %	0.0028	0.0008	0.0012	0.0044	0.0007	0.1713

r: regression coefficient. SE: standard error. 95% CI: 95% confidence interval

*TG* triglycerides, *LDL-C* low-density lipoprotein cholesterol, *Non − HDL-C* non − high-density lipoprotein cholesterol, *HDL-C* high-density lipoprotein cholesterol, *HDL* high-density lipoprotein, *LDL* low-density lipoprotein, *VLDL* very low-density lipoprotein, *PAG* polyacrylamide gel

^*^Statistical analysis by least squares method

### AUC, sensitivity, specificity, and cut-off points for non–HDL-C, TG/LDL-C ratio, and other lipid markers for LDL-MI determination

**Fig. 2:**
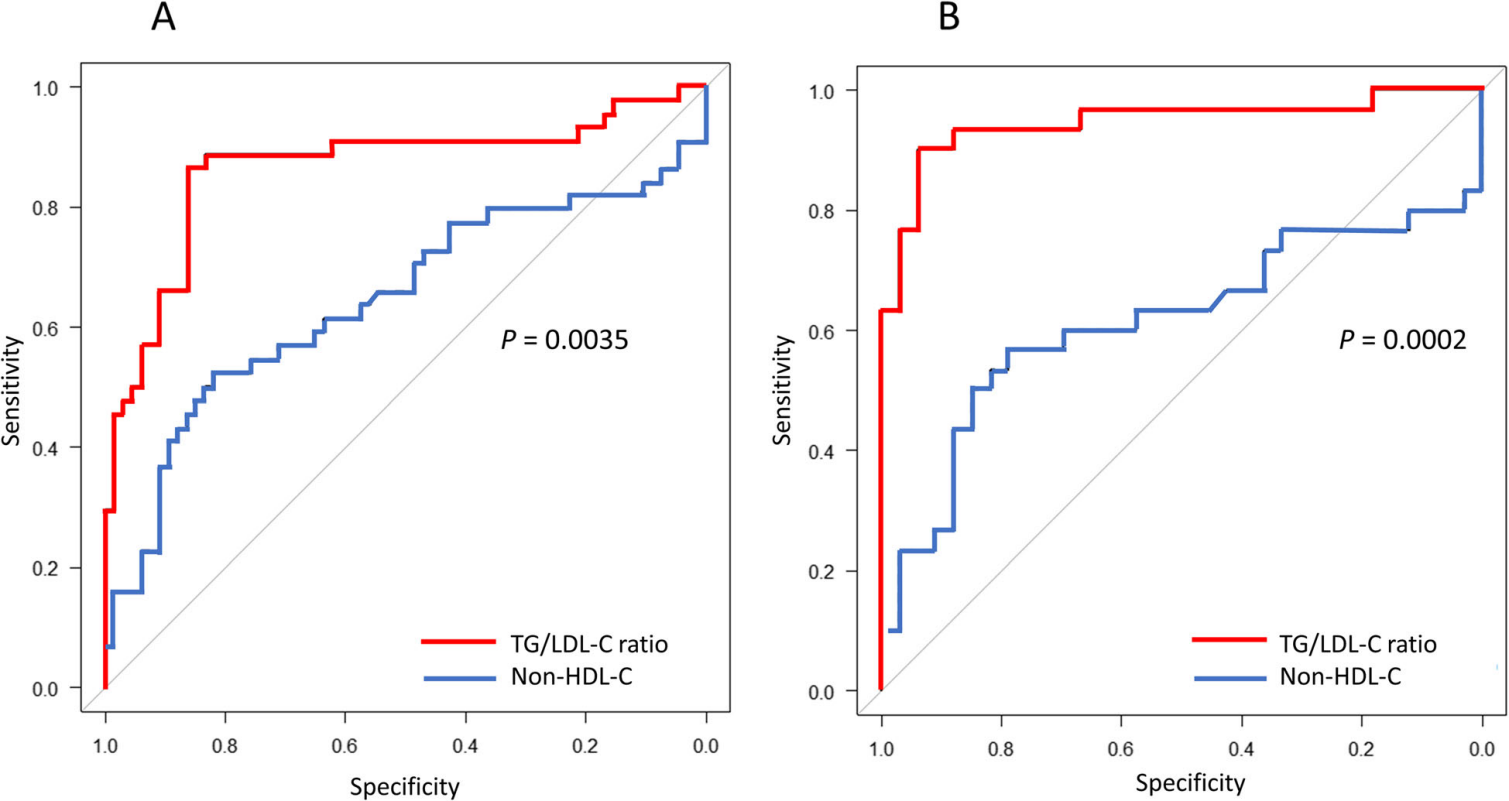
ROC curves for TG/LDL-C ratio and non–HDL-C for predicting high LDL-MI (≥ 0.400) among all patients (**A**) and those not treated with statins (**B**). AUCs of TG/LDL-C ratio (red line) and non–HDL-C (blue line) among all patients (**A**) and those not treated with statins (**B**). The AUC of the TG/LDL-C ratio was significantly greater than that of non–HDL-C among all patients (*P* = 0.0035, *χ*^*2*^ test), and those not treated with statins (*P* = 0.0002). ROC, receiver operating characteristic; LDL-MI, LDL migration index; AUC, area under the curve; TG, triglyceride; LDL-C, low-density-lipoprotein cholesterol; non–HDL-C, non–high-density-lipoprotein cholesterol

ROC curves were generated to evaluate the discriminatory ability of 9 lipid markers for LDL-MI, and the AUCs were then calculated (Table 3). Among all patients with TG ≥ 1.7 mmol/L analyzed using LDL-MI ≥ 0.400 as the gold standard, the AUC for TG/LDL-C ratio (0.865 [0.786-0.945]) was the highest among the 9 lipid markers examined. The optimal cut-off points for TG/LDL-C ratio, non–HDL-C, TG/HDL-C ratio, and non–HDL-C/HDL-C ratio for increased LDL-MI were 1.1, 4.9 mmol/L, 2.6, and 3.8, respectively. The sensitivity of the TG/LDL-C ratio for predicting higher sd-LDL was calculated as 86.4%, with a specificity of 86.4%. Both the sensitivity and specificity of the TG/LDL-C ratio were higher than those of non–HDL-C (52.3 and 81.8%, respectively), TG/HDL-C ratio (75.0 and 75.8%, respectively), and non–HDL-C/HDL-C ratio (68.2 and 68.2%, respectively). Figure 2A shows ROC curves for TG/LDL-C ratio and non–HDL-C among all study patients, and there was significant difference in the AUC between the TG/LDL-C ratio and non–HDL-C (*P* = 0.0035).

**Table 3:** Comparison of AUC, sensitivity, and specificity for each marker in predicting higher LDL-MI

Variables	AUC (95% CI)	Sensitivity	Specificity	Cut-off point
**Total (*n*** **= 110)**				
TG	0.854 (0.783-0.926)	79.5%	72.7%	3.1 mmol/L
LDL-C^a^	0.658 (0.543-0.773)	61.4%	71.2%	2.8 mmol/L
Non − HDL-C	0.643 (0.528-0.758)	52.3%	81.8%	4.9 mmol/L
LDL fraction (PAG)^a^	0.766 (0.679-0.854)	70.5%	66.7%	41%
Midband fraction (PAG)	0.738 (0.644-0.832)	75.0%	65.2%	17%
TG/LDL-C ratio	0.865 (0.786-0.945)	86.4%	86.4%	1.1
TG/HDL-C ratio	0.844 (0.772-0.917)	75.0%	75.8%	2.6
LDL-C/HDL-C ratio	0.516 (0.398-0.635)	47.7%	62.1%	2.2
Non − HDL-C/HDL-C ratio	0.717 (0.615-0.818)	68.2%	68.2%	3.8
**With statins (*n*** **= 47)**				
TG	0.755 (0.604-0.907)	71.4%	72.7%	3.1 mmol/L
LDL-C^a^	0.466 (0.262-0.671)	57.1%	57.6%	2.8 mmol/L
Non − HDL-C	0.682 (0.500-0.863)	64.3%	69.7%	4.5 mmol/L
LDL fraction (PAG)^a^	0.656 (0.491-0.821)	64.3%	66.7%	41%
Midband fraction (PAG)	0.824 (0.695-0.952)	78.6%	75.8%	21%
TG/LDL-C ratio	0.697 (0.507-0.887)	78.6%	78.3%	1.1
TG/HDL-C ratio	0.747 (0.602-0.892)	78.6%	63.6%	2.3
LDL-C/HDL-C ratio	0.623 (0.448-0.799)	64.3%	63.6%	2.4
Non − HDL-C/HDL-C ratio	0.691 (0.527-0.854)	64.3%	69.7%	3.5
**Without statins (*n*** **= 63)**				
TG	0.910 (0.839-0.981)	80.0%	87.9%	3.3 mmol/L
LDL-C^a^	0.760 (0.630-0.889)	63.3%	90.9%	2.6 mmol/L
Non − HDL-C	0.614 (0.463-0.765)	56.7%	78.8%	5.0 mmol/L
LDL fraction (PAG)^a^	0.838 (0.735-0.929)	63.3%	90.9%	35%
Midband fraction (PAG)	0.701 (0.571-0.831)	70.0%	69.7%	17%
TG/LDL-C ratio	0.945 (0.884-1.000)	90.0%	93.9%	1.1
TG/HDL-C ratio	0.892 (0.815-0.969)	80.0%	78.8%	2.7
LDL-C/HDL-C ratio	0.591 (0.435-0.747)	53.3%	81.8%	2.2
Non − HDL-C/HDL-C ratio	0.724 (0.590-0.859)	66.7%	81.8%	4.3

*CI* confidence interval, *TG* triglycerides, *LDL-C* low-density lipoprotein cholesterol, *Non-HDL-C* non-high-density lipoprotein cholesterol, *HDL-C* high-density lipoprotein cholesterol, *PAG* polyacrylamide gel

^a^the lipid markers decrease with the increase of LDL-MI

Among patients analyzed after grouping by statin use, the AUC for TG/LDL-C ratio (0.945 [0.884-1.000]) in patients not treated with statins was higher than the AUCs for the other lipid markers in patients with or without statin treatment. Figure 2B shows ROC curves for TG/LDL-C ratio and non–HDL-C in patients not treated with statins, and there was significant difference in the AUC between TG/LDL-C ratio and non–HDL-C (*P* = 0.0002). In patients not treated with statins, the optimal cut-off points for TG/LDL-C ratio, non–HDL-C, TG/HDL-C ratio, and non–HDL-C/HDL-C ratio for increased LDL-MI were 1.1, 5.0 mmol/L, 2.7, and 4.3, respectively. The sensitivity of the TG/LDL-C ratio for predicting higher sd-LDL was calculated as 90.0%, with a specificity of 93.9%. Both the sensitivity and specificity of the TG/LDL-C ratio were higher than those of non–HDL-C (56.7 and 78.8%, respectively), TG/HDL-C ratio (80.0 and 78.8%, respectively), and non–HDL-C/HDL-C ratio (66.7 and 81.8%, respectively). In patients treated with statins, the optimal cut-off points for TG/LDL-C ratio, non–HDL-C, TG/HDL-C ratio, and non–HDL-C/HDL-C ratio for increased LDL-MI were 1.1, 4.5 mmol/L, 2.3, and 3.5, respectively. The sensitivity of TG/LDL-C ratio (78.6%) was the same as that of the midband and TG/HDL-C ratio but higher than that of either non–HDL-C (64.3%) or non–HDL-C/HDL-C ratio (64.3%). There was no difference in the cut-off point for the TG/LDL-C ratio for high LDL-MI between patients with and without statin treatment. Although the cut-off points for other lipid markers for higher LDL-MI varied widely depending on statin treatment status, the TG/LDL-C ratio remained constant at approximately 1.1.

## Discussion

The present study demonstrated for the first time the usefulness of the TG/LDL-C ratio as a predictive marker for higher sd-LDL in type 2 diabetes patients with hypertriglyceridemia. In statin-free patients, the assumed cut-off point for TG/LDL-C ratio was 1.1, and the sensitivity and specificity of the ratio as a predictive marker for higher sd-LDL surpassed those of non–HDL-C or other lipid markers. TG/LDL-C ratio is the first formula proposed and considered suitable for evaluation of sd-LDL and TG-rich lipoproteins. TGs were positively correlated with LDL-MI, and LDL fraction (PAG) and LDL-C were negatively correlated with LDL-MI. The TG/LDL-C ratio more reliably predicts an increase in LDL-MI (sd-LDL). Even if LDL-C is low or within the normal range, it is possible to predict high values of sd-LDL by calculating the relative ratio with TGs. The reciprocal of this formula, LDL-C/TG ratio, was reported by Yoshida et al. and suggested as being related to sd-LDL [18]. In the present study, TG level was used as the numerator for the purpose of emphasizing the existence of TG-rich apoB-containing lipoproteins.

Specific clinical data collected in routine clinical practice can be combined with other related data to increase clinical usefulness for the diagnosis or estimation of various diseases [19]. The routine lipid panel consists of LDL-C, HDL-C, TGs, and total cholesterol. Several additional parameters, such as non–HDL-C (total cholesterol minus HDL-C), LDL-C/HDL-C ratio [20], non–HDL-C/HDL-C ratio [21], and TG/HDL-C ratio [22], are emerging as valuable adjuncts to the standard panel. Non–HDL-C is used to evaluate apoB-containing lipoproteins and sd-LDL [23]. In particular, increased non–HDL-C concentration is reportedly associated with residual risk for CVD and has been adopted as a guideline for lipid management [7]. In general, when non–HDL-C increases, the cholesterol contained in TG-rich lipoprotein increases, as do total cholesterol concentrations [24]. In patients with hypertension and/or insulin resistance, the metabolism of lipoproteins is delayed, and they remain in the blood circulation for a variety of reasons [25]. Cholesterol-rich and TG-rich apoB-containing remnant lipoproteins are taken up by macrophages, and cholesterol accumulates in atherosclerotic lesions [26, 27].

Non–HDL-C is easily calculated by subtracting HDL-C from total cholesterol. Non–HDL-C can provide a better risk estimation compared with LDL-C, in particular in hypertriglyceridemia combined with diabetes, metabolic syndrome, or chronic kidney disease. This is supported by a recent meta-analysis including 14 statin trials, 7 fibrate trials, and 6 nicotinic acid trials [28]. Non–HDL-C is used as an estimation of the total number of atherogenic particles in plasma (VLDL + intermediate-density lipoprotein + LDL) and relates well to apoB levels. Non–HDL-C may be greatly affected by LDL-C and apoB concentrations [29]. A high correlation exists between the changes in non–HDL-C and TGs [30].

In a previous study, it was found that type 2 diabetes patients with hypertriglyceridemia can be divided into two groups: those with relatively low LDL-C and those with normal or high LDL-C. In the former group, TGs and sd-LDL were also higher than those in the latter group, but there was no difference in non–HDL-C between the two groups (4.9 [4.0-5.4] mmol/L vs. 4.8 [4.5-5.2] mmol/L) [9]. Alternatively, the increase in TGs and decrease in LDL-C could have been synchronized [13, 31], and the change in non–HDL-C might have been offset. The non–HDL-C measurement formula evaluates the cholesterol levels of TGs and LDL, which are rich in apoB. An increase in sd-LDL reflecting hypertriglyceridemia and a decrease in large, buoyant LDL might occur simultaneously in type 2 diabetes patients. Patients with type 2 diabetes and/or insulin resistance have increased production of VLDL1, a larger-sized VLDL [32, 33]. VLDL1, after the action of cholesteryl ester transfer protein, produces cholesterol-poor LDL particles (sd-LDL) via hydrolysis of TGs by hepatic TG lipase. Simultaneously, the normal size of VLDL is reduced and the large LDL particles produced by the VLDL metabolic process are reduced [33]. As evidence, this study showed that both the LDL-C and LDL fractions in PAG electrophoresis are negatively correlated with LDL-MI. If so, calculated non–HDL-C, regardless of the LDL-C assay method used, will largely reflect the decline in buoyant LDL present in some type 2 diabetes patients, and it is estimated that the increase in non–HDL-C would not be as expected.

**Fig. 3:**
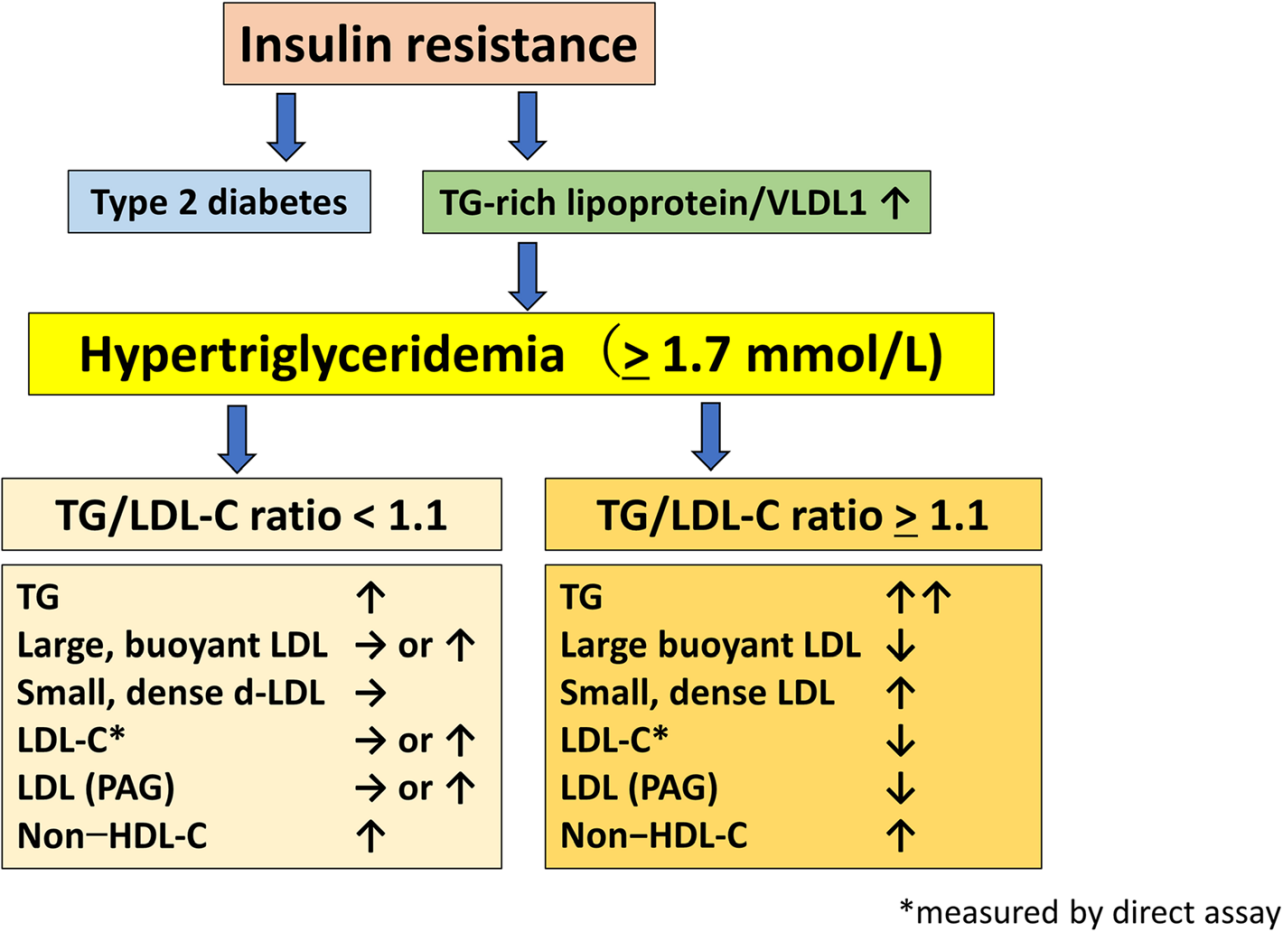
Graphical summary of the main findings of the present study. TG, triglyceride; LDL-C, low-density-lipoprotein cholesterol; non–HDL-C, non–high-density-lipoprotein cholesterol; PAG, polyacrylamide gel electrophoresis

The changes in LDL composition would affect the measurement of non–HDL-C and thus impair its clinical reliability, and these changes might also affect the relationship between non–HDL-C and sd-LDL [9]. In contrast, another lipid marker that indirectly compares the changes in sd-LDL and buoyant LDL (i.e., the TG/LDL-C ratio) seems to more accurately reflect the pathophysiology of dyslipidemia in type 2 diabetes patients. A new subset of atherogenic lipoproteins consisting of LDL-C and TGs is proposed, with LDL-C and TGs serving as surrogates for LDL/intermediate-density lipoprotein and VLDL, respectively. The TG/LDL-C ratio reflects LDL and VLDL and can be a new predictive marker for sd-LDL increase. The fact that the cut-off point for the TG/LDL-C ratio was constant irrespective of statin treatment suggests that the TG/LDL-C ratio is universal and reliable for the prediction of sd-LDL increase. Figure 3 visually summarizes the findings of the present study.

The REDUCE-IT trial showed that treatment with icosapent ethyl significantly reduced CVD events without any change in non–HDL-C in patients with CVD risk and increased baseline TGs but well-controlled LDL-C [30, 34]. The REDUCE-IT trial results may alter the approach to the management of hypertriglyceridemic patients whose lipid phenotype requires more intensive treatment beyond LDL-C lowering alone.

### Comparisons with other studies and what the current work adds to existing knowledge

Yoshida et al. first proposed the LDL-C/TG ratio and total cholesterol (TC)/TG ratio and reported that these ratios were useful as predictors of increased sd-LDL (substituted by LDL-MI) [18]. There are only two reports of formulas for lipid assessment using TG and LDL-C, including the present report. Although patients with normolipidemia were included and no information about glucose tolerance was available, it was reported that the sensitivity and specificity of the TC/TG ratio for predicting high sd-LDL were 72.7 and 83.6% [18], respectively, values lower than those in present study. In a study involving 994 non-diabetic patients with TG ≤ 4.5 mmol/L (400 mg/dL), the AUC of non–HDL-C was 0.871 (0.840-0.901), and the sensitivity, specificity and positive predictive value for predicting high sd-LDL (≥ 46 mg/dL via direct assay) were 78.8, 79.8 and 54.9%, respectively [35]. In that study, LDL-C was calculated using Friedewald’s formula. A simple comparison between the previous and present studies reveals that the TG/LDL-C ratio of the present study is superior in terms of AUC, sensitivity, and specificity versus non–HDL-C of the previous study for the prediction of high sd-LDL. If the usefulness of the TG/LDL-C ratio in predicting CVD is widely recognized, the recommendations of the atherosclerosis guidelines may need to be revised [7].

### Study strengths and limitations

There are several strengths to this study. First, a reliable LDL-C direct assay, Metabolead LDL-C® (Hitachi Kasei Diagnostic Systems), was used for LDL-C estimation, and the results were consistent with the lipoprotein PAG electrophoresis results [9]. Moreover, this direct method has already been shown to be consistent with ultracentrifugation, unless TGs exceed 11.3 mmol/L [15, 16]. There were no patients with a fasting TG level of ≥11.3 mmol/L in this study; therefore, any effect of hypertriglyceridemia on the LDL-C assay could be ruled out [36, 37]. A study observing changes in LDL (measured by high-performance liquid chromatography) before and after pemafibrate reported an increase in LDL, especially large, buoyant LDL, in addition to a decrease in sd-LDL after the treatment [38]. Conversely, it indicated that there was a relative reduction in baseline large, buoyant LDL in some patients with hypertriglyceridemia [13]. This is consistent with the decreases in baseline LDL-C (measured via the direct method) and baseline LDL fraction in PAG electrophoresis. Second, PAG electrophoresis, which is a simple and inexpensive method, was used for the estimation of sd-LDL and lipoprotein fractions.

This study also has several limitations. First, instead of directly measuring sd-LDL, determination of LDL-MI by PAG electrophoresis was used as a substitute. However, many reports have indicated that the results of both are strongly correlated [13, 14]. Second, the study design was retrospective; therefore, the reliability of the findings could be inferior comparing with studies of differing design. Third, there were relatively few target patients in this study. Statistically significant results were obtained despite the small number of patients in present study, however. Future studies should enroll a larger number of target patients. Fourth, the prevalence of CVD in the target patients in the present study was low, and it was not possible to evaluate CVD due to quantitative changes in the TG/LDL-C ratio or LDL-MI. Fifth, there may be some specificity in the clinical background of the patients included in present study. There was a high proportion of male patients. Patients treated with statins were older and had lower eGFR. It is generally known that Okinawan subjects (who were analyzed in the present study) tend to develop obesity from an early age and that the frequencies of dyslipidemia and metabolic syndrome in these subjects are high [39, 40]. It is therefore unclear whether the results of this study are universally applicable to all Japanese or other ethnic groups. Finally, this study did not show results for serum apolipoproteins such as apoB, apolipoprotein E and lipoprotein(a) [Lp(a)]. Lp(a) is considered a genetic risk factor for CVD, especially in young people [41]. However, the patients in this study were older and the involvement of Lp(a). in CVD was presumed to be low.

## Conclusion

In the ACCORD lipid study, combination therapy with a statin and fibrate did not demonstrably suppress CVD events in patients with type 2 diabetes [42]. However, in diabetes patients with high TGs and low HDL-C, the onset of CVD was persistently suppressed even after the ACCORD trial was completed [43]. The TG/LDL-C ratio may offer a simple clinical tool for predicting increased sd-LDL. The TG/LDL-C ratio was useful in the pathologic evaluation of type 2 diabetes patients with high TGs compared with the recommended measurement of non–HDL-C. Even in statin-treated type 2 diabetes patients whose LDL-C remains within the therapeutic range, clear increases in TG/LDL-C ratio and sd-LDL with hypertriglyceridemia are inherent, and pemafibrate or ezetimibe, which have a sd-LDL-lowering effect, might be considered as drugs for add-on therapy [9, 44]. In addition, statin dosage adjustment may be necessary because of the expected increase in LDL-C associated with the increase in large, buoyant LDL after treatment with these drugs. Assessment of the patient’s condition using the TG/LDL-C ratio and appropriate treatment selection may reduce the residual risk of CVD. Further review of the diagnostic indices and control standards for lipids in patients with type 2 diabetes is warranted. A new large-scale clinical trial currently underway is evaluating the usefulness of TG-lowering therapy for suppressing the development of CVD [45]. The usefulness of the TG/LDL-C ratio for CVD prediction may also be demonstrated in that trial.

## Data Availability

The data that support the findings in this study are available from Medical Plaza Daido Central. Data are available from the authors upon reasonable request and with permission of Medical Plaza Daido Central.
